# The comparative effectiveness and cost-effectiveness of ranibizumab for neovascular macular degeneration revisited

**DOI:** 10.1186/s40942-016-0058-3

**Published:** 2017-02-13

**Authors:** Gary C. Brown, Melissa M. Brown, Heidi B. Lieske, Adam Turpcu, Yamina Rajput

**Affiliations:** 1 The Center for Value-Based Medicine®, Box 6181, Hilton Head, SC 29938 USA; 2 The Eye Research Institute, Philadelphia, PA USA; 3 The Retina Service, Wills Eye Institute, Jefferson Medical College, Philadelphia, PA USA; 4 The Research Department, Wills Eye Institute, Jefferson Medical College, Philadelphia, PA USA; 5 The Department of Ophthalmology, Emory University School of Medicine, Atlanta, GA USA; 6 The Healthcare Economics Unit, Genentech, Inc., South San Francisco, CA USA

**Keywords:** Ranibizumab, Cost-utility analysis, Age-related macular degeneration, Clinical cost-utility model

## Abstract

**Background:**

To compare a near decade of follow-up, newer control cohort data, use of both the societal and third party insurer cost perspectives, and integration of unilateral/bilateral therapy on the comparative effectiveness and cost-effectiveness of intravitreal ranibizumab therapy for neovascular, age-related macular degeneration (AMD).

**Methods:**

Value-Based Medicine^®^, 12-year, combined-eye model, cost-utility analysis employing MARINA and HORIZON clinical trial data. Preference-based comparative effectiveness outcomes were quantified in (1) QALY (quality-adjusted life-year) gain, and (2) percent improvement in quality-of-life, while cost-effectiveness outcomes were quantified in (3) the cost-utility ratio (CUR) and financial return-on-investment (ROI) to society.

**Results:**

Using MARINA and HORIZON trial data and a meta-analysis control cohort after 24 months, ranibizumab therapy conferred a combined-eye patient value (quality-of-life) gain of 16.3%, versus 10.4% found in 2006. The two-year direct ophthalmic medical cost for ranibizumab therapy was $46,450, a 33.8% real dollar decrease from 2006. The societal cost perspective CUR was −$242,920/QALY, indicating a $282,517 financial return-on-investment (ROI), or 12.3%/year to society for direct ophthalmic medical costs expended. The 3rd party insurer CUR ranged from $21,199/QALY utilizing all direct, medical costs, to $69,591/QALY using direct ophthalmic medical costs.

**Conclusions:**

Ranibizumab therapy for neovascular AMD in 2015, considering treatment of both eyes, conferred greater patient value gain (comparative effectiveness) and improved cost-effectiveness than in 2006, as well as a large monetary return-on-investment to the Gross Domestic Product and nation’s wealth. The model herein integrates important novel features for neovascular age-related macular degeneration, vitreoretinal cost effectiveness analyses, including: (1) treatment of both eyes, (2) a long-term, untreated control cohort, and (3) the use of societal costs.

## Background

Intravitreal ranibizumab therapy has been shown to be an effective treatment for minimally classic/occult [1, 2] and classic [3] subfoveal choroidal neovascularization occurring with neovascular age-related macular degeneration (NVAMD) in well designed and executed Level 1 [4] clinical trials. Ranibizumab (Lucentis^®^) is a recombinant humanized, IgG_1_ kappa isotype, monoclonal antibody fragment designed for intraocular use [5]. It binds to and inhibits the biologic activity of human vascular endothelial growth factor A (VEGF-A), a molecule believed to be primarily responsible for the development of the choroidal neovascularization associated with NVAMD.

A number of healthcare economic studies, including cost-utility analyses, cost-effectiveness analyses and cost-benefit analyses, have addressed the use of ranibizumab for the treatment of NVAMD [6–12]. Unfortunately, even with sensitivity analyses [13], very few cost-utility analyses (also called cost-effectiveness analyses by some [13]) are comparable due to the use of different utilities, dissimilar utility respondents, differing cost perspectives, diverse costs bases, unlike discounting and so forth [14]. The Center for Value-Based Medicine^®^ has estimated that over 27,000,000 different, input variable combinations can be used in a single cost-utility analysis [4]. Even one different input can make a major difference in the outcome [4, 14].

Value-Based Medicine^®^ (VBM) [4, 14–17] is a methodology of cost-utility analysis employing standardized inputs to determine the *patient value gain* and *financial value gain* conferred by healthcare interventions. Patient value gain is quantified by improvement in length-of-life and/or quality-of-life; it is measured in QALY (quality-adjusted life-year) gain and percent patient value gain. The intervention which confers the greatest patient value has the greatest *comparative effectiveness.* VBM integrates *financial value gain* using the resources expended for the *patient value gain* in terms of the cost-utility ratio ($/QALY, or dollars expended per QALY gained), and the dollar return-on-investment (ROI) to society for the interventional, direct medical costs expended (cost-benefit ratio). VBM [4, 14–17] standardizes cost-utility analysis variables by typically utilizing: (1) time tradeoff utilities, (2) patient utility respondents, (3) the average national Medicare Fee Schedule, and (4) both societal and 3rd party insurer cost perspectives.

The Panel on Cost-Effectiveness in Health and Medicine [13, 18] has recommended that cost-utility analyses use the societal cost perspective. While the literature is replete with cost-utility analyses touting the cost-effectiveness of healthcare interventions, most do not address the entirety of societal costs affected by healthcare interventions [14].

Beauchamp and colleagues [19] have long advocated that medicine’s business is the production of *patient value* and *economic return*. We agree that healthcare economic analyses should ideally provide accurate assessments of, not only conferred patient value and the societal cost-utility, but also the financial ROI accrued to society and to the GDP (Gross Domestic Product) [14].

In 2008, we published a third party insurer, second-eye model, cost-utility analysis (using 2006 data and costs) on ranibizumab for the treatment of neovascular AMD utilizing MARINA (Minimally classic/occult trial of the Anti-VEGF antibody Ranibizumab In the treatment of Neovascular Age-Related Macular Degeneration) Study data [9]. Longer treatment data, improved control cohort data [20] and societal cost data [21, 22], however, have since become available that dramatically alter the *patient value gain. Financial ROI* was not addressed in our 2008 report [9]. Thus, we believe it important to present these data in a cost-utility analysis demonstrating recent *patient value gain* and *financial value gain* for ranibizumab therapy for AMD. These will be compared with 2006 data [9] that are available.

## Methods

### The MARINA and HORIZON trials

The MARINA Trial was a Phase III, 24-month, randomized clinical trial comparing intravitreal ranibizumab therapy with a 0.3 mg dose, a 0.5 mg dose, or sham therapy for neovascular AMD. The study parameters and cost-utility analysis assumptions utilized herein are shown in Table 1. We did not analyze 0.3 mg data since the 0.5 mg dose was the one approved for use by the Food and Drug Administration [5].

**Table 1:** MARINA study clinical and cost-utility analysis parameters

Clinical features [1]	
Each participant had minimally classic or occult, subfoveal choroidal neovascularization	
Best corrected ETDRS entrance vision in the affected eye: 20/40–20/320	
Choroidal neovascular lesions <12 disc areas at baseline	
Baseline vision: mean 20/80 − 1 in both the ranibizumab treatment and sham treatment cohorts	
Mean baseline age: 77 years	
Treatment protocol: Participants were randomized equally to: (1) a 0.5 mg intravitreal ranibizumab dose cohort (n = 240), (2) a 0.3 mg intravitreal ranibizumab dose cohort (n = 238) or (3) a sham injection treatment cohort (n = 238)	
Only data from the 0.5 mg ranibizumab cohort (0.5 mg was the dose eventually approved by the Food & Drug Administration [5]) and the sham treatment control cohort were utilized in the cost-utility analysis herein	
The average participant received 22 × 0.05 cc intravitreal injections, given approximately monthly, over 2 years	
Cost-utility analysis assumptions	
Mean life expectancy: 12 years for the control and ranibizumab study cohorts [9]	
12-year time span for model utilizing 2-year MARINA data from the sham treatment control group and the 0.05 mg ranibizumab treatment group	
Combined-eye model [14, 15]	
Societal and 3rd party insurer cost perspectives	
Cost basis: average 2015, national, Medicare Fee Schedule	
Vision utilities (based upon visual acuity in the better-seeing eye) [24–31]	
Vision	Utility
20/20 OU	0.97
20/40	0.80
20/80	0.701
20/200	0.62
20/640	0.538
Sham treatment, control cohort data utilized: mean vision in MARINA Study for years 1 and 2; Lineweaver-Burke plot meta-analysis [20] control cohort for years 3–12	
Treatment cohort (0.5 mg ranibizumab) mean vision: MARINA Study for years 1 and 2, HORIZON open-label extension trial for 25–49 months, LOCF (last observation carried forward) for months 49–144 [23]	
Net Present Value (NPV) analysis discounts value outcomes and costs at a 3% annual rate, as per the Panel on Cost-Effectiveness in Health and Medicine [13, 18]	
Adverse events as previously listed [9]	
Patient utilities as previously listed [9]	

*MARINA* minimally classic/occult trial of the Anti-VEGF antibody ranibizumab in the treatment of neovascular age-related macular degeneration (AMD), *ETDRS* early treatment diabetic retinopathy study

At randomization, mean vision in the MARINA Trial was 20/80 − 1 in both the 0.5 mg ranibizumab cohort and sham control cohort. By the end of 24 months, a mean 22 intravitreal ranibizumab injections were administered in the treatment cohort [1, 9]. At this time, the HORIZON (Open-Label Extension Trial of Ranibizumab for Choroidal Neovascularization Secondary to Age-Related Macular Degeneration) extension study of the MARINA Trial was utilized to model the ranibizumab treatment cohort from 25 to 48 months, after which a LOCF (last observation carried forward) methodology was used [23]. A mean 3.6 of injections were given from months 25 to 48. The timeline of the cost-utility model was 12 years, the average life expectancy of a participant in the MARINA Trial [1, 9]. The mean visions at different time intervals are shown in Table 2.

**Table 2:** Mean MARINA study vision levels (years 1–2), HORIZON extension trial (years 3–4), then last observation carried forward to year 12 in the 0.5 mg treatment cohort, with a meta-analysis control cohort from years 3–12

Time	Control cohort	0.5 mg treatment cohort
Baseline	20/80 − 1	20/80 − 1
6 months	20/100 − 2	20/63
12 months	20/126	20/63 + 1
18 months	20/160 + 2	20/63 + 1
24 months	20/160 − 1.5	20/63 + 1
3 years	20/250	20/63 + 1
4 years	20/320	20/80 + 2
5 years	20/400	20/80 + 1
6 years	20/500 + 2	20/80 + 1
7 years	20/500	20/80 + 1
8 years	20/640 + 2	20/80 + 1
9–12 years	20/640	20/80 + 1

### Control cohort

Sham cohort, MARINA trial [1], control data were used for 24 months, after which MARINA control cohort eyes were eligible for treatment with ranibizumab. Thus, control data for years 3–12 were derived from the meta-analysis of Shah and Del Priore [20], who used a double reciprocal (Lineweaver-Burke plot) methodology to assess the natural course of untreated subfoveal choroidal neovascularization associated with AMD. Shah and Del Priore plotted the variables of 1/(letters lost) versus 1/(time in months) (r^2^ = .9521) for the control groups of six major subfoveal, neovascular, AMD clinical trials [20]. The longest follow-up among these trials was 84 months. Shah and Del Priore [20] found the pattern of visual loss in eyes with subfoveal neovascular AMD to be uniform across the trials, with differences arising primarily from the time of entry into a trial.

### Utilities

The time tradeoff vision utilities utilized herein, as in our previous report [9] were obtained with the approval of the Wills Eye Hospital Institutional Review Board from a cohort of over 1100 interviews of patients with ocular diseases. The utilities are reproducible and validated across age, level of education, ocular diseases, ethnicity, gender, income level, and the presence of comorbidities [24–31]. The disutilities from adverse events and their associated costs are integrated and treated in the same fashion as in our previous report [9].

### Costs

Healthcare economic costs can be subdivided into: (1) direct medical costs (physician, facility and drug costs), (2) direct non-medical costs (caregiver, residence, and activities of daily living costs), and (3) indirect costs (decreased wages) [14]. In the previous ranibizumab cost-utility analysis [9] only 2006 U.S. nominal, direct ophthalmic medical costs were utilized. The current study uses 2015 U.S. societal costs in real dollars.

#### Direct non-ophthalmic medical costs

Not included previously [9], but included herein, are data from Javitt et al. [21] on Medicare’s excess, direct, non-ophthalmic medical costs associated with vision loss. These costs are attributable to increased depression, injury, SNF (skilled nursing facility) admissions, nursing home admissions, and other unexplained Medicare costs. They were adjusted to 2015 real dollars using the Medical Care component of the Consumer Price Index (CPI) [32]. These costs saved by ranibizumab therapy totaled $54,974 (Table 3).

**Table 3:** societal costs associated with ranibizumab therapy for neovascular age-related macular degeneration

Parameter	Cost
Direct ophthalmic medical costs	
Ranibizumab therapy (81.3% bilateral)	$79,056
Direct non-ophthalmic medical costs	
Injury reduction	(−$664)
Depression reduction	(−$2543)
SNF cost decrease	(−$4100)
Unexplained medical cost decrease	(−$28,598)
Nursing home	(−$19,069)
Total offsetting costs	(−$54,974)
Total direct medical costs	$24,082
Direct non-medical costs	
Paid caregiver salaries^a^	(−$82,419)
Salary gain for freed-up caregivers now able to take up gainful employment	(−$215,123)
Total direct non-medical costs	(−$297,542)
Indirect medical costs	
Mean patient wage gain	(−$9057)
Total societal costs	(−$282,517)

All costs in 2015 U.S. real dollars, *SNF* skilled nursing facility

^a^As per Schmier et al. [22], 27.7% of caregiver costs are paid

Direct non-ophthalmic medical costs, direct non-medical costs and indirect medical costs only accrue for the second-eye model, since the first-eye model, fellow eye still has good vision. With Markov modeling (Treeage Software Inc., Williamstown, MA) and the data of Barbazetto et al. [33] 81.3% of the time during the 12-year model people have bilateral neovascular AMD (second-eye model). Thus the societal costs, excluding the direct ophthalmic medical costs, were all multiplied by 0.813. All direct ophthalmic costs are accrued, whether for the first eye or second eye treated.

#### Direct non-medical costs

Also absent in the previous report [9], but included herein, are the caregiver costs for AMD demonstrated by Schmier et al. [22] (Table 3). These were adjusted to 2015 U.S. dollars with the general CPI [22]. Schmier and colleagues [22] noted 27.7% of caregivers were paid and 72.3% were unpaid.

Salaries of previously unpaid caregivers freed-up to undertake paid employment as a result of ranibizumab therapy were treated as direct non-medical costs contributing to a gain in the GDP. The 27.7% of already paid caregiver salaries [22] decrease the GDP since these jobs are made unnecessary by better vision obtained from ranibizumab therapy. While displaced paid caregivers can obtain other jobs, we prefer a conservative analysis.

#### Indirect medical costs

The major, AMD indirect medical cost is loss of patient salary. Data from the Americans with Disabilities Household Economic Studies [34] show, between the decreased employment rate and decreased salary, a person with mild difficulty reading (vision <20/40) has an annual salary of $22,551, versus $18,915 for a person with severe difficult reading and $47,230 for a non-disabled person [35]. If a person with severe difficulty reading achieves >20/40 vision, the salary gain is ($47,230 − $18,913=) $28,317, while the gain from mild difficulty reading to >20/40 vision is ($47,230 − $22,551=) $24,679. Taking into account the proportions of people employed at different ages, the total salary gain from ranibizumab therapy was $9057.

#### Gross Domestic Product (GDP)

The GDP is a primarily a measure of the final goods and services produced within the country in a year [36]. It has four components: (1) consumer spending, (2) industry investment in new productive capabilities, (3) the excess of exports minus imports, and (4) the goods and services bought by the government. While a simplistic view, increasing the GDP increases the country’s wealth [36].

### Net present value analysis

*Net present value analysis* weighs the costs versus the QALY gain, each reduced to present value by discounting. The Panel for Cost-Effectiveness in Health and Medicine [18] has recommended the 3% annual discount rate used herein.

### Second-eye, first-eye and combined-eye models [9, 37]

The concept of first-eye and second-eye models was developed at the Center for Value-Based Medicine^®^ based upon data from patients with ocular diseases. The first-eye model assumes vision in the fellow eye is normal; interventional patient value, or benefit, is usually not conferred until fellow eye vision deteriorates. The second-eye model assumes vision in first eye has already deteriorated. Thus, patient value gain is conferred from the initiation of therapy. The combined-eye model used for our base case is a weighted average of first- and second-eye models. Most ocular cost-utility models have used the second-eye model [6–12, 15, 17], suggesting a bias toward overstatement of patient value gains and greater cost-effectiveness.

Treeage Pro Healthcare, Excel Module 2014 software (Treeage Software Inc., Williamstown, MA) was utilized to assess the value gain for ranibizumab therapy in the first-eye and combined-eye models in our Sensitivity Analysis.

## Results

### Patient value gain

With the 12-year combined-eye model, the mean base case, patient value gain from intravitreal ranibizumab therapy utilizing the meta-analysis control cohort [20] from years 3 to 12 was 1.136 QALYs. This converted to a mean 16.3% quality-of-life improvement for the average patient, versus a 10.4% quality-of-life gain in 2006 [9]. The second-eye model accrued 1.372 QALYs, a 22.8% improvement in quality-of-life, versus 15.8% in 2006 [9].

### Financial value gain

#### Direct ophthalmic medical costs

Our previous cost-utility analysis on MARINA data utilized a 3rd party insurer (direct ophthalmic medical costs) cost perspective with a 2006 average national Medicare Fee Schedule cost basis [9]. The 2006 direct ophthalmic medical cost was $52,652, while our comparable cost for the same drug and services in 2015 nominal dollars was $46,450, an 11.8% decrease since 2006. Adjusting for the Medical Care component of the CPI [32], the 2006 real dollar cost would be expected to rise to $70,161. Thus, over nine years from 2006 to 2015, adjusting with the Medical Care component of the CPI, the cost of two-year ranibizumab therapy in real dollars decreased by 33.8% (Table 4). Nonetheless, with the addition of ranibizumab treatment costs of $8120, simulating the HORIZON Trial in years 3 and 4 after baseline [23], and mean treatment costs of $24,287 for second eye conversion to neovascular AMD over 12 years [33], the total direct ophthalmic cost in 2015 is a more accurate $79,056.

**Table 4:** 2006 Versus 2015 direct ophthalmic medical costs and patient value gain versus ranibizumab therapy for first two years

Cost	2006	2015
Physician	$6167 (11.7%)	$2611 (5.6%)
Ranibizumab	$44,812 (85.2%)	$42,665 (91.9%)
Diagnostic tests	$1230 (2.3%)	$1174 (2.5%)
Post-operative antibiotics	$414 (0.8%)	$0 (0.0%)
Total	$52,652 (100%)	$46,450 (100%)
Adjusted with medical CPI to 2015 US $	$70,161	$46,450
Decrease in real dollars from 2006 to 2015	NA	−33.8%
Patient value (quality-of-life) gain	2006	2015
First-eye model	6.4%	9.8%
Second-eye model	15.8%	22.8%
Combined-eye model	10.4%	16.3%

Parentheses indicate percent of total direct ophthalmic medical costs. *NA* not applicable. Note that patient value gain = quality-of-life gain

#### Direct non-ophthalmic medical costs (Table 3)

The 12-year direct medical cost saved from the reduction of the depression, injury reduction, nursing home, SNF and so forth (Table 3) was $54,974 [21]. When this was subtracted from the direct ophthalmic medical cost of $79,056, the total direct medical cost was $24,082.

#### Direct non-medical costs. (Table 3)

The 12-year, direct non-medical cost saving for paid caregivers, who comprise 27.7% of caregivers, was $82,419 (Table 3). The $215,123 for unpaid caregivers corresponded to potential salary earned by freed-up, unpaid caregivers obtaining paid jobs [35]. This accrues to society and the GDP (Table 3).

#### Indirect medical costs: increased patient salaries (Table 3)

The 12-year cost associated with higher employment rates and improved salaries for ranibizumab-treated neovascular AMD patients with improved vision was $9057.

#### Total societal costs

The sum of costs accruing against the direct ophthalmic medical costs was (−$361,573). When the total direct ophthalmic medical cost of $79,056 was subtracted, the resultant dollars returned to society (or negative costs), primarily patients, was (−$282,517) (Table 3).

#### Physician fees

Physician fees in 2006 comprised 11.7% of direct ophthalmic medical costs and in 2015 comprised 5.6%. Ranibizumab costs in 2006 comprised 85.2% of costs, versus 91.9% of total costs in 2015 (Table 4).

#### Gross Domestic Product

The costs which contribute to the GDP are shown in Table 5 [36, 37]. Direct ophthalmic medical costs increase the GDP, but the direct non-ophthalmic medical costs obviated by ranibizumab therapy decrease the GDP. With the societal cost perspective, the overall contribution to the GDP over the 12 years of the model was $165,842, a 7.0% annual ROI referent to the $79,056 in direct medical costs expended for ranibizumab therapy. With the 3rd party insurer cost perspective, there was a negative ROI for the direct ophthalmic medical costs expended (Table 5).

**Table 5:** Societal and 3rd party insurer cost perspective cost-utility analyses (2015 U.S. real dollars discounted at 3% annually)

Cost perspective	Societal	3rd party insurer
Ranibizumab therapy, direct ocular medical costs^a^	79,056	79,056
Depression^b^	(2543)	(2543)
Injury^b^	(664)	(664)
Subacute Nursing Facility (SNF)^b^	(4100)	(4100)
Nursing home^b^	(19,046)	(19,046)
Unexplained direct medical Medicare costs^b^	(28,598)	(28,598)
Patient employment gain^a^	(9057)	–
Paid caregivers released from jobs^b^	(82,419)	–
Increased paid employment by freed-up unpaid caregivers^a^	(215,123)	–
Total costs offsetting the direct ophthalmic medical costs	(361,573)	(54,974)
Direct medical costs + offsetting costs = financial return to society	282,517	(24,082)
Financial ROI for direct ophthalmic medical costs expended	14.7%	−3.5%
QALY gain: combined-eye model	1.136	1.136
QALY gain: second-eye model	1.372	1.372
VRQOL gain, combined-eye model	16.3%	16.3%
VRQOL gain, second-eye model	22.8%	22.8%
$/QALY, combined-eye model	(242,921)	20,707
$/QALY, second-eye model	(205,916)	17,552
Addition to GDP	165,842	24,082
Financial ROI for GDP referent to direct ophthalmic medical costs expended	7.0%	−11.4%

Costs in parentheses () are negative costs, indicating they are accrued against the direct ophthalmic medical costs of ranibizumab therapy

*MARINA* minimally classic/occult trial of the anti-VEGF antibody ranibizumab in the treatment of neovascular age-related macular degeneration (AMD) [9], *VRQOL* vision-related quality-of-life, *3rd party insurer* third party insurer cost perspective utilizing the direct ocular medical costs and the direct non-ophthalmic medical costs

*ROI* return-on-investment, *QALY* quality-adjusted life-year, *$/QALY* dollars expended per QALY gained, gained, *VRQOL* vision-related quality-of-life

^a^Costs adding to the Gross Domestic Product (GDP)

^b^ Costs subtracting from the Gross Domestic Product

### Cost-utility analysis

An *average* cost-utility analysis compares an intervention to no therapy, while an *incremental* cost-utility analysis compares an intervention to other interventions [14].

#### Average CUR

The average, combined-eye, CUR with the MARINA Study societal cost perspective was (−$282,517/1.163=) (−243,921/QALY) (Table 5). A negative CUR indicates a dominant therapy in that it is less expensive than the alternative (observation) and at the same time it generates greater patient value than the alternative (observation herein). The CUR associated with the 3rd party insurer cost perspective using all direct medical costs (ophthalmic + non-ophthalmic) was ($24,082/1.136=) $21,199/QALY. With direct ophthalmic medical costs alone, the CUR was ($79,056/1.136=) $69,591/QALY. While the latter costs for NVAMD therapy over 12 years were higher than in 2006 [9], the earlier model did not include HORIZON trial and fellow eye treatment costs.

#### Incremental CURs

Incremental CURs are calculated from Value-Based Medicine^®^, published cost-utility data [15, 17] using a 3rd party insurer cost perspective (Table 6). The incremental CUR for ranibizumab therapy for NVAMD versus laser therapy was $20,643/QALY, while that referent to intravitreal pegaptanib was $3546/QALY and for photodynamic therapy was $16,044/QALY, all very cost-effective.

**Table 6:** Incremental cost-utility ratios of ranibizumab referent to laser photocoagulation, intravitreal pegaptanib, and PDT

Intervention	QALY gain	Cost	Avr. $/QALY	Incr. $/QALY^a^
Ranibizumab, 2015	1.372	$24,082	$17,552	NA
Laser	0.25717	$1071	$6157	$20,643
Pegaptanib, intravitreal	0.83417	$22,175	$26,589	$3546
PDT	0.74715	$14,057	$18,818	$16,044

All cost-utility analyses in this table use patient utilities, a 3rd party insurer cost perspective and 2nd-eye model. *PDT* photodynamic therapy with verteporfin, *QALY* dollars expended per quality-adjusted life year gained, or cost-utility ratio, Avr. *$/QALY* average cost-utility ratio, Incr. *NA* not applicable, *ranib.* ranibizumab therapy. All outcomes and costs are discounted at 3% annually

^a^Incremental cost-utility ratio of ranibizumab referent to the other interventions

### Sensitivity analysis

The sensitivity analyses were performed using the societal cost perspective, average cost-utility, and a 3% annual discount rate unless otherwise indicated (Table 7).

**Table 7:** Sensitivity analysis results for ranibizumab for the treatment of subfoveal neovascular AMD utilizing a societal cost perspective, average cost-utility analysis (bilateral treatment model unless otherwise specified)

Interventional variables	QALY/QOL gain	Cost	$/QALY
Eye model			
2nd eye model, societal costs	1.372/22.8%	(−$282,517)	(−$205,971)
2nd eye model, 3rd party insurer costs	1.372/22.8%	$24,082	$17,557
1st eye model, societal costs	0.680/9.8%	(−$282,517)	(−$415,367)
1st eye model, 3rd party insurer costs	0.680/9.8%	$24,082	$35,406
Combined-eye model, societal costs—BASE CASE	1.136/16.3%	(−$282,517)	(−$248,639)
Combined-eye model, all 3rd party insurer costs	1.136/16.3%	$24,082	$21,194
Combined-eye model, direct ophthalmic medical costs only	1.136/16.3%	$79,056	$69,592
Costs			
Ranibizumab cost increased 100%	1.136/16.3%	(−$233,271)	(−$205,344)
Four additional ranibizumab annually, years 3–13	1.136/16.3%	(−$213,910)	(−$188,301)
Caregiver costs excluded	1.136/16.3%	$15,025	$13,226
Cost of therapy decreased by 50%	1.136/16.3%	(−$322,045)	(−$283,490)
Treat-and-extend regimen costs (assuming same value gain) [38]^a^			
Direct ophthalmic medical costs only, treat-and- extend, no fellow eye treatment costs	1.136/16.3%	$52,250	$45,995
Current study, direct ophthalmic medical costs, no fellow eye treatment costs	1.136/16.3%	$54,569	$48,036
Value gain			
Patient value gain (QALY gain) drops by 50% for years 5–12, societal costs	0.688/9.9%	(−$282,517)	(−$410,757)
Patient value gain (QALY gain) drops by 50% years 5–12, 3rd party insurer costs	0.688/9.9%	$24,082	$35,013
Cost-utility			
For cost-utility of $50,000/QALY	1.136/16.3%	$56,800	$50,000
For cost-utility of $100,000/QALY	1.136/16.3%	$113,600	$100,000

*Parentheses ()* negative dollars and negative cost-utility ratios, *QALY* quality-adjusted life year, *QOL* quality-of-life, *$/QALY* cost-utility ratio, or dollars expended per QALY gained. Note that a negative cost-utility ratio simply means that ranibizumab therapy dominates sham therapy in that it confers greater patient value and is less expensive

^a^Ranibizumab injections [38] = year 1—7.6, year 2—5.7, year 3—5.8, year 4—1.8, year 5 forward—none

#### Eye models

The societal cost perspective, first-eye model conferred 0.680 QALY, a 9.8% improvement in quality-of-life, while the second-eye model conferred 1.372 QALYs, a 22.8% improvement in quality-of-life. The first-eye model, societal, CUR was (−$415,367)/QALY, while the second-eye model, societal, CUR was (−$205,971)/QALY. Since the costs were the same with each of these three variants, a less negative CUR indicates greater cost-effectiveness. In general, however negative CURs are not comparable, but rather indicate dominance.

#### Changes in cost

Increasing ranibizumab costs by 100%, the combined-eye, adding four additional ranibizumab injections annually and excluding caregiver costs all yielded cost-effective results (Table 7). Using costs from a treat-and-extend model [38], the 3rd party insurer cost perspective had a CUR of $45,995, similar to that of $48,036 using comparable costs in our current analysis.

#### Value gain

When the QALY gain decreased by 50% from years 5 to 12, the CUR was (−$462,960/QALY).

#### $50,000/QALY

For a societal cost perspective CUR of $50,000/QALY, the direct ophthalmic medical costs had to be increased to $339,317, a 329% increase over the current $$79,056. For a CUR of $100,000/QALY, the direct ophthalmic medical costs had to be increased to $432,021, a 401% increase over $79,056.

### Comparisons

A comparison of patient value conferred by other drug classes, using a similar cost-utility model, shows the considerable value gain from ranibizumab therapy. Data from the Center for Value-Based Medicine^®^ are shown in Table 8 [13, 16, 37, 39, 40]. Among the drug groups, only anti-depressants, β-blockers for glaucoma, and proton pump inhibitors match or exceed the patient value gain conferred by ranibizumab therapy for NVAMD. Ranibizumab value gain far exceeds that conferred by interventions for systemic arterial hypertension, hyperlipidemia, insomnia, erectile dysfunction, osteoporosis and allergy.

**Table 8:** Patient value conferred by drug classes across medicine [16, 37]

Drug class	Indication	# of drugs	Patient value gain (%)
Histamine H-1 receptor antagonists, oral	Seasonal allergy	10	4–7
Histamine H-1 receptor antagonists, topical ocular	Allergy, ocular	6	0.1–9.9
SSRI drugs	Depression, major	5	20–23
Anti-depressants, non-SSRI	Depression, major	6	21–32
cGMP-specific phosphodi-esterase inhibitors	Erectile dysfunction	3	2.7–2.9
Proton pump inhibitors	Acute erosive esophagitis	5	13–26
Proton pump inhibitors	Gastroesophageal reflux	5	8–14
Proton pump inhibitors	Zollinger-Ellison Syndrome	5	29–38
Histamine H-2 receptor antagonists	Acute erosive esophagitis	4	5–11
Histamine H-2 receptor antagonists	Gastroesophageal reflux	4	3–7
B-blockers, topical	Glaucoma	3	16–19
Statins	Hyperlipidemia	6	3–5
Diuretics	Hypertension, systemic arterial	5	7.7–9.4
ACE inhibitors	Hypertension, systemic arterial	9	6.5–9.1
B-blockers, oral	Hypertension, systemic arterial	7	6.3–9.1
Hypnotics	Insomnia	7	1.2–8.8
*Ranibizumab, combined eye model*	*Neovascular age*-*related macular degeneration, current study*	*1*	*16.3*
Bisphosphonates	Osteoporosis	3	0.8–1.1
α1-adrenergic blockers	Prostatic hyperplasia	3	0.6–1.4

All values are from the Center for Value-Based Medicine Pharmaceutical Value Index database©

*SSRI* selective serotonin reuptake inhibitor, *statins* HMG-CoA reductase inhibitors

## Discussion

### Patient value

Our analysis demonstrates that ranibizumab therapy for NVAMD confers considerable patient value. This does not refer to cost, but rather the improvement in quality-of-life conferred by ranibizumab. Our previous analysis of patient value gain for the same intervention [9] demonstrated a 10.4% combined-eye model, value gain [9], versus 16.3% in the current analysis. With HORIZON trial data [23] and control cohort data from Shah and DelPriore [20], our 2015 analysis provides more reliable data than our 2006 data [9], which used a LOCF methodology from month 25 forward. It should be noted that ophthalmic patient utility gains tend to be similar in the United States, Canada and Europe [14, 24, 41, 42]. Utilities appear to be innate to human nature rather than one society [14]. Thus, the patient value gain data in this analysis can be integrated with the associated costs for ranibizumab therapy for NVAMD in another country and likely provide a valid cost-utility analysis for that country.

### Costs

As used in the MARINA Study [1], intravitreal ranibizumab therapy yields a considerable financial return-on-investment to society and the GDP. The GDP is often regarded as a determinant of a nation’s wealth [36]. Ranibizumab therapy improves physical well-being, or human capital, which directly results in financial capital gain and also allows a dramatic decrease in caregiver costs.

The societal costs associated with many healthcare interventions are not well-defined, and only recently have those associated with vision loss and macular degeneration been identified [22, 23, 37, 39, 40, 43]. The majority of cost-utility analyses have not included caregiver costs, but they are especially relevant for untreated bilateral NVAMD [23].

Concerning employment and the decreased salary associated with disabilities, Americans with Disabilities Household Economic Studies data from the U.S. Census Bureau [34] are enlightening. Those with difficulty reading have lower mean earnings than those with hearing difficulty, a learning disability or in a wheelchair [34].

### Cost-utility

The societal cost perspective generally produces an improved CUR for an intervention compared to the direct ophthalmic medical cost perspective [9, 37, 40]. This concept is demonstrated herein for ranibizumab (Table 5). Caregiver costs saved, improved salary and employment and direct medical costs obviated (depression, injury, facility admissions etc.) herein all accrue against the direct ophthalmic medical costs expended.

#### Cost-effectiveness standards

Healthcare interventions costing <$100,000/QALY are generally accepted as cost-effective in the U.S. [44] The World Health Organization [45] suggests an upper cost-effectiveness limit of 3× GDP per capita, or ~$164,000/QALY for the United States [46]. The use of ranibizumab in the societal cost perspective is very cost-effective by each of these criteria.

### Potential study weaknesses

As with any analysis, there are potential inherent weaknesses in this study.

#### Neovascular AMD sub-types

The MARINA clinical trial assessed occult and minimally classic AMD variants, which together comprise 80% of neovascular AMD cases [47]. Ranibizumab therapy for classic neovascular AMD (20% of cases), as studied in the ANCHOR clinical trial [3], results in slightly greater, patient value gain than in the MARINA trial [1], suggesting the data in our analysis are actually conservative.

#### Absence of long-term clinical trial data

The absence of randomized clinical trial data after two years is a drawback, through HORIZON data provide treatment efficacy thru 48 months and Shah and DelPriore provide an excellent long-term control cohort [20]. The SEVEN-UP Study [48] followed ranibizumab-treated NVAMD patients for up to seven years, but the very small numbers of patients (~10% of those enrolled) likely introduce bias. Thus, we did not employ these data. The sensitivity analysis, however, demonstrates excellent cost-effectiveness even if ranibizumab loses 50% of its efficacy from months 49 to 144.

#### Unpaid caregiver costs

Unpaid caregivers may not obtain paid employment once freed-up to do so, but the sensitivity analysis addressed this as well. Even when all costs related to unpaid caregivers gaining paid employment are excluded, the CUR is still very cost-effective at $13,226/QALY.

#### Use of other drugs

Our intent herein was to compare the changes in preference-based comparative effectiveness and cost-utility for the same drug treating the same disease over a near decade. We thus did not perform cost-utility analyses on other VEGF inhibitors used for the treatment of NVAMD, such as aflibercept and bevacizumab.

### Study strengths: differences from 2006 to 2015

Our paper utilized patient utilities and standardized VBM format [14]. In addition, four major changes were integrated into the current study that differentiate the results from our 2006 study costs [9]. These include: (1) a more reliable control cohort from Shah and DelPriore [20], (2) HORIZON extension trial treatment data [23], (3) societal costs from the studies of Javitt [21], Schmier [22] and Steinmetz [34], and (4) costs associated with the conversion of fellow eyes with atrophic AMD to NVAMD over the 12-year model time frame [33]. We are unaware that these unique parameters have been used together by other authors for assessing the cost-effectiveness of neovascular AMD therapy, especially in conjunction with primary utilities obtained from over 1100 ophthalmic patients. We believe they create a much more robust model than those used previously for the cost-effectiveness associated with treatment of NVAMD. As advances occur in healthcare interventions, they also take place in the economic evaluation of interventions. In this regard, it can be seen that a one-year cohort of 178,000 new neovascular AMD cases [49, 50] treated with ranibizumab will return over $50 billion to society over a 12-year period (Table 9).

**Table 9:** Macroeconomic (ROI) for ranibizumab therapy for neovascular AMD using the societal cost perspective, combined-eye model

Cost perspective	Direct ophthalmic medical cost per patient	Annual # of cases	Total direct ophthalmic medical costs	Net cost/case with direct ophthalmic costs	12-year financial gain
MARINA—costs off-setting the s direct ocular medical costs	$79,056	178,000	$14.1 billion	(−$282,517)	$50.2 billion
MARINA GDP gain	$79,056	178,000	$14.1 billion	(−$165,842)	$29.5 billion

*ROI* return-on-investment referent to the direct medical costs associated with ranibizumab administration, *GDP* Gross Domestic Product, *MARINA* minimally classic/occult trial of the anti-VEGF antibody ranibizumab in the treatment of neovascular age-related macular degeneration (AMD)

## Conclusions

In summary, the 2015 patient value gain conferred by, and costs associated with, ranibizumab therapy for NVAMD compare favorably with those from 2006. Longer-term treatment cohort follow-up, a more reliable control cohort, the inclusion of societal costs and a bilateral treatment model all result in a more robust 2015 analysis. The therapy provides great patient value than many common interventions across healthcare and also provides a considerable financial return-on-investment to society Changing patterns of therapy, such as increased use of a treat-and-extend model may result in more favorable cost-effectiveness yet for ranibizumab therapy.

## Abbreviations

VBM: Value-Based Medicine
AMD: age-related macular degeneration
NVAMD: neovascular age-related macular degeneration
GDP: Gross Domestic Product
LOCF: last observation carried forward
CUR: cost-utility ratio
ROI: return on investment
MARINA: minimally classic/occult trial of the anti-VEGF antibody ranibizumab in the treatment of neovascular age-related macular degeneration
VEGF: vascular endothelial growth factor
CPI: consumer price index
